# Impact of DJ-1 and Helix 8 on the Proteome and Degradome of Neuron-Like Cells

**DOI:** 10.3390/cells10020404

**Published:** 2021-02-16

**Authors:** Ursula Kern, Klemens Fröhlich, Johanna Bedacht, Nico Schmidt, Martin L. Biniossek, Nicole Gensch, Katja Baerenfaller, Oliver Schilling

**Affiliations:** 1 Institute of Surgical Pathology, Medical Center, University of Freiburg, Breisacher Straße 115a, D-79106 Freiburg, Germany; ursula.kern@uniklinik-freiburg.de (U.K.); klemens.froehlich@mol-med.uni-freiburg.de (K.F.); 2 Institute of Molecular Medicine and Cell Research, Faculty of Medicine, University of Freiburg, 79110 Freiburg, Germany; johanna.bedacht@uniklinik-freiburg.de (J.B.); nico.schmidt@merkur.uni-freiburg.de (N.S.); martin.biniossek@mol-med.uni-freiburg.de (M.L.B.); 3 Faculty of Biology, University of Freiburg, 79104 Freiburg, Germany; 4 Spemann Graduate School of Biology and Medicine, University of Freiburg, 79104 Freiburg, Germany; 5 Core Facility Signalling Factory, BIOSS Centre for Biological Signalling Studies, University of Freiburg, 79104 Freiburg, Germany; nicole.gensch@bioss.uni-freiburg.de; 6 Swiss Institute of Allergy and Asthma Research (SIAF), University of Zurich, and Swiss Institute of Bioinformatics (SIB), 7265 Davos, Switzerland; katja.baerenfaller@siaf.uzh.ch

**Keywords:** PARK7, parkinson disease, neurodegenerative disease, protease, glycase, TAILS, proteomics, degradation, lysosome, cathepsin b

## Abstract

DJ-1 is an abundant and ubiquitous component of cellular proteomes. DJ-1 supposedly exerts a wide variety of molecular functions, ranging from enzymatic activities as a deglycase, protease, and esterase to chaperone functions. However, a consensus perspective on its molecular function in the cellular context has not yet been reached. Structurally, the C-terminal helix 8 of DJ-1 has been proposed to constitute a propeptide whose proteolytic removal transforms a DJ-1 zymogen to an active hydrolase with potential proteolytic activity. To better understand the cell-contextual functionality of DJ-1 and the role of helix 8, we employed post-mitotically differentiated, neuron-like SH-SY5Y neuroblastoma cells with stable over-expression of full length DJ-1 or DJ-1 lacking helix 8 (ΔH8), either with a native catalytically active site (C106) or an inactive site (C106A active site mutation). Global proteome comparison of cells over-expressing DJ-1 ΔH8 with native or mutated active site cysteine indicated a strong impact on mitochondrial biology. N-terminomic profiling however did not highlight direct protease substrate candidates for DJ-1 ΔH8, but linked DJ-1 to elevated levels of activated lysosomal proteases, albeit presumably in an indirect manner. Finally, we show that DJ-1 ΔH8 loses the deglycation activity of full length DJ-1. Our study further establishes DJ-1 as deglycation enzyme. Helix 8 is essential for the deglycation activity but dispensable for the impact on lysosomal and mitochondrial biology; further illustrating the pleiotropic nature of DJ-1.

## 1. Introduction

Loss-of-function mutations in the gene PARK7, encoding the 20-kDa protein DJ-1, are associated with autosomal recessive, early-onset Parkinson‘s disease (PD) [1]. The assumed neuroprotective functions of DJ-1 have been attributed mainly to its effect on mitochondrial maintenance and anti-oxidant properties [2]. However, the biochemical mechanism how DJ-1 protects cells against oxidative stress and mitochondrial damage is still unclear. In numerous studies, different DJ-1 activities have been reported, including: transcriptional regulation by protein stabilization [3,4,5], RNA binding and translational repression [6], cellular redox sensor (reviewed in [7]), peroxidase [8], chaperone [9,10,11], glutathione-independent glyoxalase [12], esterase [13], deglycase [14] and protease activity [15,16,17,18]. DJ-1 is a 189 amino acid, dimeric, evolutionary conserved protein (reviewed in [19]). It shares sequence homology with the PfpI family of bacterial intracellular proteases and with heat shock protein 31 (Hsp31), an *Escherichia coli* chaperone with protease activity. The crystal structure shows that DJ-1 contains a putative catalytic nucleophile Cys-106 (C106), which has the potential to form a Cys-His catalytic diad with His-126 [20,21]. However, the C-terminal alpha helix H8 appears to block access of substrates to the putative catalytic site. Weak C106-dependent proteolytic activity of purified DJ-1 was reported using casein as a substrate [18]. In vitro casein cleavage was higher in a DJ-1 truncation mutant lacking the C-terminal 15 amino acid peptide containing alpha helix H8, and the authors concluded that DJ-1 converts from a zymogen to an active protease by cleavage of H8 [15]. DJ-1 also showed C106-dependent catalytic activity when incubated with a peptide library with a clear preference for valine in P1 and alanine in P1’ at the cleavage site [17]. Two substrates, c-abl oncogene 1 product and kinesin family member 1B, were suggested in this study. In cells, in contrast to biochemical in vitro systems, protease activity and access to substrates is tightly regulated to prevent fatal damage to proteins. The identification of natural protease substrates is crucial to understanding the role of a protease in a specific physiologic context. Here we aimed to identify natural neuronal DJ-1 proteolytic substrates in human neuron-like cells using N-terminomics [22] as well as to probe the deglycase activity of DJ-1. We did not observe protease substrates that appear to be directly cleaved by DJ-1. However, our findings implicate DJ-1 in the regulation of lysosomal proteolysis. In addition, we confirm that DJ-1 protects cells from protein glycation. Helix 8 is essential for the deglycation activity but dispensable for the impact on lysosomal biology.

## 2. Materials and Methods

### 2.1. Vectors and Cell Transduction

Human DJ-1 (Ensembl: ENSG00000116288, MIM:602533) I.M.A.G.E. cDNA clone IRATp970A044D was used for site directed mutagenesis and generation of the following four different DJ-1 constructs: full length DJ-1 with wild-type C106; full length DJ-1 with active site mutated C106A; DJ-1 lacking helix 8 (C-terminal 15 residues) with wild-type C106; DJ-1 lacking helix 8 (C-terminal 15 residues) with active site mutated C106A. DJ-1 variants were cloned into a bicistronic pMIG expression vector containing an internal ribosomal entry site (IRES) and GFP allowing stoichiometric expression of untagged DJ-1 variants. A three plasmid system was used for the generation of high titer retroviral particles for SH-SY5Y transduction [23]. Successfully transduced cells were selected with 800 µg/ml G418 for three weeks. Subpopulations of each new cell line expressing the four different DJ-1 variants or harboring the empty vector were selected by GFP-based fluorescence-assisted cell sorting (FACS) using a BD Biosciences FACS Aria flow cytometer. To confirm successful genomic integration of the respective DJ-1 constructs, genomic DNA (gDNA) of the established cell lines was isolated using a gDNA extraction kit (Qiagen), DJ-1 gDNA was amplified by PCR with a forward primer binding the vector backbone after the 5′LTTR: TACACCCTAAGCCTCCGCCT and a reverse primer binding in the DJ-1 sequence: AGGCCCCCGGCTTGTAAGA and sequenced with the sequencing primer: CCCTTGAACCTCCTCGTTCGACC.

### 2.2. Cell Culture and Differentiation

SH-SY5Y cells were purchased from LGS standards. Cells were grown in standard Dulbecco’s Modified Eagle Medium DMEM/F12, Gibco, Thermo Fischer) supplemented with 10% fetal calf serum, 1% L-glutamine and 1% penicillin/streptomycin on standard plastic cell culture dishes in a sterile incubator (37 °C, 5% CO2). For differentiation a previously published protocol for the generation of a homogenous population of fully differentiated, neurotrophic factor-dependent human neuron-like cells [24] was used with minor modifications: SH-SY5Y cells were seeded at an initial density of 10^4 cells/cm^2^ on Advanced cell culture dishes (Greiner). On the following three days 10 µM all-trans retinoic acid (RA) was added in standard medium every 24 h. After three days in the presence of RA, the cells were washed twice with DPBS and grown in serum-free medium supplemented with 50 ng/ml Brain Derived Neurotrophic Factor (BDNF) for four days.

### 2.3. Cell Proliferation Measurement

Real-time cell proliferation was assessed using the xCelligence^®^ System (Roche, Basel Switzerland). Two thousand cells per well were seeded in E-plates and differentiated as described above. Proliferation was measured in 1 h intervals during the course of differentiation. A BrdU ELISA (Roche Basel Switzerland) was used to determine proliferation after 7 days of differentiation according to the manufacturer‘s protocol. Briefly, 1 × 10^4^ cells per well were seeded in a 96 well plate and differentiated as described above. At day 6 of the differentiation protocol BrdU was added for 24 h before fixation of the cells and immuno-detection and quantification of BrdU incorporation after 30 min incubation with anti-BrdU-POD solution and 30 min incubation with substrate solution. The absorbance of the samples was measured at 370 nm (reference wavelength: 492 nm).

### 2.4. Sample Preparation for Quantitative Proteomics

Sample preparation for liquid chromatography–tandem mass spectrometry (LC-MS/MS)-based proteome comparison was essentially performed as described previously [25] including denaturation and alkylation, trypsin digestion, stable isotopic labeling and pre-fractionation via strong cation exchange chromatography (SCX, replicates 1 and 2) or high pH reversed phase chromatography (hpH-RP, replicate 3).

Differentiated SH-SY5Y DJ-1^WT^ΔH8 and SH-SY5Y DJ-1^C106A^ΔH8 cells were washed three times with phosphate buffered saline (PBS), harvested with a cell scraper, and lysed on ice in lysis buffer (50 mM Tris pH 7.5, 150 mM NaCl, 1% NP-40, 1% sodium deoxycholate, 0.02% SDS, 1 mM EDTA) with protease inhibitors (10 µM E64d, 1 mM PMSF and 5 mM EDTA) added fresh to avoid protein degradation during lysis. Lysates were heat incubated for five min at 95 °C prior to ultra-sonication for 10 × 30 s. For protein precipitation, cell lysates were incubated with nine volumes of ice-cold acetone and one volume of ethanol for one hour at −80 °C. After centrifugation at 4500 g, protein pellets were washed three times with ice-cold ethanol and reconstituted in 100 mM NaOH using ultra-sonication, followed by pH adjustment to 8.0 with HEPES. For the proteome comparison, samples were labeled by reductive dimethylation of primary amines either “light” (formaldehyde CD2O + sodiumcyanoborohydrid NaBH3CN) or “heavy” (formaldehyde 13CD2O + sodium cyanoborodeuteride NaBD3CN). The label was switched in the second experiment. Samples were pre-fractionated on C18 Hypersep columns using 15%, 25% and 60% acetonitrile in 1% ammonium hydroxide as elution buffers respectively.

### 2.5. Sample Preparation for N-Terminomics

The Terminal Amine Isotopic Labeling of Substrates (TAILS) protocol was modified from [26,27]. Cells were lysed using 5 mM TCEP, 0.1% RapiGest, 100 mM HEPES pH 8.0 and samples were immediately heat denatured for 10 min at 95 °C. Subsequently, DNA was sheared using ultra-sonication for 10 × 30 s. Iodoacetamide (20 mM) was added for alkylation of cysteines and incubated for 30 min at room temperature in the dark. α- and ε-amines were labeled for 18 h at room temperature by adding tandem mass tag (TMT) 11 plex reagents (Thermo), which were resuspended in acetonitrile with a protein to TMT reagents mass ratio of 1:8. Following pooling of samples, sequencing grade trypsin (Worthington) was added for digestion in a trypsin to protein mass ratio of 1:50 and the sample was incubated for 2 h at 50 °C. The same amount of trypsin was added again and the sample was incubated for 18 h at 37 °C. Internal peptides were then coupled to an aldehyde-functionalized polymer in the presence of 50 mM NaBH3CN and removed from TMT-blocked N-terminal peptides by ultrafiltration through a 10 kDa MWCO filter (Millipore, Burlington, MA, USA). The flow-through, containing TMT-blocked N-terminal peptides, was desalted using C18 columns (Hypersep, Thermo, Waltham, MA, USA). The sample was pre-fractionated on C18 Hypersep columns using 15%, 25% and 60% acetonitrile in 1% ammonium hydroxide as elution buffers, respectively. For exact ratio determination of Cathepsin B propeptide cleavage in four independent TAILS pilot experiments was analyzed, which were performed as described previously [28].

### 2.6. LC-MS/MS

LC-MS/MS of the global proteome comparison was performed with a Q Exactive PLUS System (Thermo Fisher) coupled to an Easy nLC 1000 (Thermo Fisher) as described previously [29].

LC-MS/MS of the TMT TAILS experiment was measured on an Orbitrap Eclipse mass spectrometer (Thermo Fisher) coupled to an Easy nLC 1200 (Thermo Fisher). Pre-columns with 100 µm ID were self-packed with 3µm C18 AQ (Dr. Maisch) to a length of 2 cm. A 75 µm Picofrit column (New Objective) was self-packed with 1.9 µm C18 AQ (Dr. Maisch) to a length of 20 cm. Buffer A consisted of 0.1% formic acid, buffer B consisted of 80% acetonitrile in 0.1% formic acid. The samples were separated using a 70 min linear gradient from 10% to 38% B followed by a 5 min linear gradient from 38% to 47% buffer B. The mass spectrometer was operated in data dependent acquisition mode with a TMT MS2 quantitation method. A survey scan from 400–1600 m/z at 120 K resolution was followed by MS2 events up to 2 s. Standard precursor filter options from the TMT MS2 method editor node were used. For MS2 scans, peptides were fragmented using higher energy collision dissociation (HCD) with CE 38, maximum injection time 54 msec at 30 K resolution with TMT and TMTpro resolution enhancement activated.

### 2.7. Proteomic Data Analysis

For proteome comparisons, raw files were analyzed using the default settings of MaxQuant [30] Version 1.6.0.16 except for the following parameter changes: The labels DimethylLys4, DimethylNterm4 and DimethylLys8, DimethylNterm8 were set. iBAQ and no variable modifications were chosen and protein quantification was allowed for label min ratio count 1. The data were compared to a complete human reviewed database without isoforms downloaded from UniProt in June 2017 containing 20188 entries. For proteome comparisons the parameters: enzyme Trypsin, digestion mode specific (0 missed cleavages) and “re-quantify” were chosen.

For the TMT TAILS analysis MaxQuant Version 1.6.12.0 was used. Reporter ion MS2 with TMT11 plex and semi-tryptic enzyme specificity (ArgC) were chosen. TAILS data were analyzed by filtering semi‑tryptic peptides, more specifically, no lysine or arginine prior to the peptide sequence were allowed and initiator methionines were also excluded. Subsequently, data were z-score normalized and linear models for microarray data (LIMMA package from R) was used to detect differentially abundant peptides. Cleavage motif analysis was performed using an online tool for protease specificity characterization [31]. The Dimethylation TAILS experiments were analyzed using MaxQuant Version 1.6.14.0. Standard Quantitation with appropriate multiplicity was chosen depending on the experimental design: DimethNterm0, 4 or 8 and DimethLys0, 4 or 8 and semi-tryptic enzyme specificity (ArgC) was chosen.

Downstream analyses were performed using Perseus [32] and R, employing linear models and differential expression for microarray data (LIMMA) [33] for the detection of differentially abundant proteins and peptides. Gene ontology enrichment analyses were performed using topGO [34]. Visualizations were created by using tidyverse [35] RColorBrewer [36] GraphPad Prism, or PVD [37].

### 2.8. Western Blot

Whole cell lysates were prepared as described for quantitative proteome comparison. Cell lysates were centrifuged at 15000 g for 2 min, and protein concentration in the supernatant was determined with a bicinchoninic acid protein assay (BCA, Pierce). Equal amounts of protein extracts (5 to 40 μg depending on the antibody used) were resolved by SDS-PAGE and transferred onto polyvinylidene fluoride (PVDF) membranes using a semi-dry blotting system (Bio-Rad, Hercules, CA, USA). For immune detection of proteins, membranes were probed with the following antibodies: DJ-1 (R&D Systems 3668, Minneapolis, MN, USA), GAP43 (Böhringer 1379011, Ingelheim, Germany), β3-Tubulin (Sigma T8660, Kavasaki, Japan), Cathepsin B (R&D Systems AF953), methylglyoxal (MGO, Cell Biolabs Inc. STA-011, San Diego, CA, USA). β-Tubulin (Sigma Aldrich T6199) or GAPDH (abcam 9484) were used as loading control. Methylglyoxal (MGO) immunoblots were stripped by incubating the membrane with pre-heated stripping buffer (2% SDS, 62.5 mM Tris-HCl pH 6.8, 1:125 *v/v* ß-mercaptoethanol) at 50 °C for 45 min, followed by rinsing for 1–2 h with water and 5 min with TBST before they were blocked and re-probed with GAPDH antibody. Signal intensity was analyzed with ImageJ [38].

### 2.9. Protein Glycation

Differentiated SH-SY5Y cells were incubated with 5 mM methylglyoxal (MGO) for 2 h under normal cell culture conditions. Cells were washed with DPBS three times and whole cell lysates were prepared as described for Western Blot. Protein glycation was probed by immunoblotting for MGO as described above.

## 3. Results

### 3.1. In Vitro System of Differentiated SH SY5Y Neuron-Like Cells

**Figure 1:**
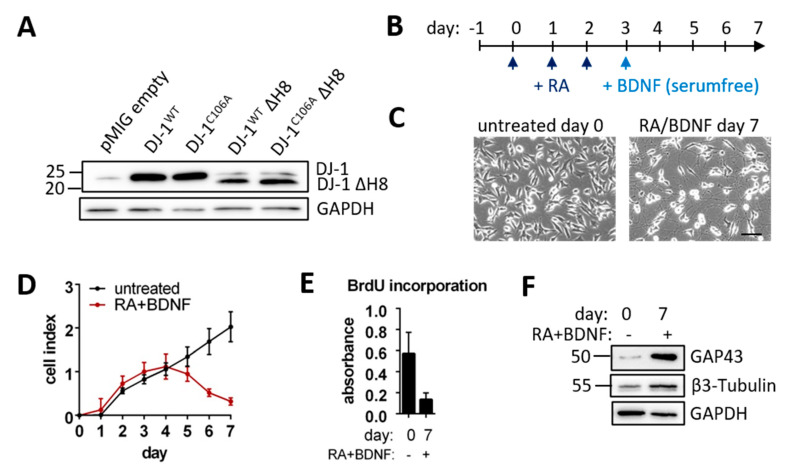
**Established cell system:** (**A**) DJ-1 Immunoblot of SH-SY5Y cells overexpressing different DJ-1 variants or pMIG empty vector (**B**) Timeline for SH-SY5Y differentiation with retinoic acid (RA) and brain derived neurotrophic factor (BDNF) (**C**) Micrographs of undifferentiated and differentiated SH-SY5Y cells (scale bar: 100 µm) (**D**) Real-time SH-SY5Y cell proliferation over 7 days of RA + BDNF treatment depicted as cell index over time of three technical replicates was assessed by absorbance at 370 nm (**E**) BrdU incorporation of untreated and RA + BDNF treated SH-SY5Y cells measured as the absorbance at 370 nm wavelength. (**F**) Immunoblots of mature neuronal markers Growth-associated Protein 43 (GAP43) and β-3-Tubulin with GAPDH as loading control.

To probe for putative, natural neuronal DJ-1 proteolytic substrates we overexpressed different DJ‑1 variants (Figure S1A) in the human neuroblastoma cell line SH-SY5Y with a comparatively low background of endogenous DJ-1 (Figure 1A). The putative active protease form of DJ-1, DJ-1^WT^ΔH8, lacks the 15 C-terminal amino acid residues 175–189 forming helix 8 (H8), which can block substrate access to the catalytic site. In the inactivated form, DJ-1^C106A^ΔH8, the proposed catalytic nucleophile cysteine-106 (C106) was mutated to alanine (Figure S1B). For comparison, full length DJ-1^WT^ and DJ-1^C106A^ and the empty vector were overexpressed as well. All variants were expressed with an IRES-GFP. Subpopulations of transduced cells with equal GFP signal intensity were selected using FACS, and five different cell lines with stable overexpression of the different DJ-1 variants or the empty vector were established. Although the same gates were used for the sorting of all cell lines, higher protein expression of the full length variants in comparison to the truncated variants was observed (Figure 1A). The different overexpression levels need to be taken into consideration when directly comparing the impact of full length DJ-1 and DJ-1ΔH8 on cellular behavior.

We consider differentiated SH-SY5Y cells to be a more adequate model than non-differentiated cells to investigate cell-physiological roles of proteins such as DJ-1; as highlighted by a proteome study comparing undifferentiated and differentiated SH-SY5Y cells, which substantiated profound differences of their respective proteome biology [39]. To this end, we differentiated the aforementioned transduced SH-SY5Y cell lines stably over-expressing different DJ-1 variants, using a protocol adapted from [24] including three days of sequential retinoic acid (RA) and subsequently four days of Brain Derived Neurotrophic Factor (BDNF) treatment (Figure 1B). Untransduced differentiated SH-SY5Y cells displayed formation and extension of neurite-like structures (Figure 1C) and dramatically reduced proliferation as shown by real-time monitoring and BrdU incorporation (Figure 1D,E). We observed a higher expression of the mature neuronal markers Growth-associated Protein 43 (GAP43) and β3-Tubulin following differentiation (Figure 1F). Hence, we refer to these cells as neuron-like, post-mitotically differentiated cells. Each transduced cell line could be differentiated into neuron-like cells with neurite-like structures (Figure S1C).

### 3.2. Degradome Analysis

To detect putative proteolytic substrates of DJ-1 and to assess the influence of DJ-1 on the cellular degradome, we performed TAILS, an N-terminomic technique for the identification and quantification of native and proteolytically generated protein N-termini [22]. In brief, protein N-termini and N-termini generated by proteolytic cleavage are chemically protected and tagged with a tandem mass tag (TMT). Proteins are then subjected to tryptic digestion. The thereby generated Neo N-Termini are not chemically protected and can be depleted using an amine‑reactive polymer. We compared differentiated SH-SY5Y cells overexpressing the catalytically active form DJ-1^WT^ΔH8 to cells overexpressing the catalytically inactive form DJ-1^C106A^ΔH8 in three replicates. We chose an 11plex tandem mass tag (TMT)-based labeling approach. We identified a total of 2223 peptides, which either represent native protein N-termini or proteolytically generated N‑termini (Supplementary Table S1).

**Figure 2:**
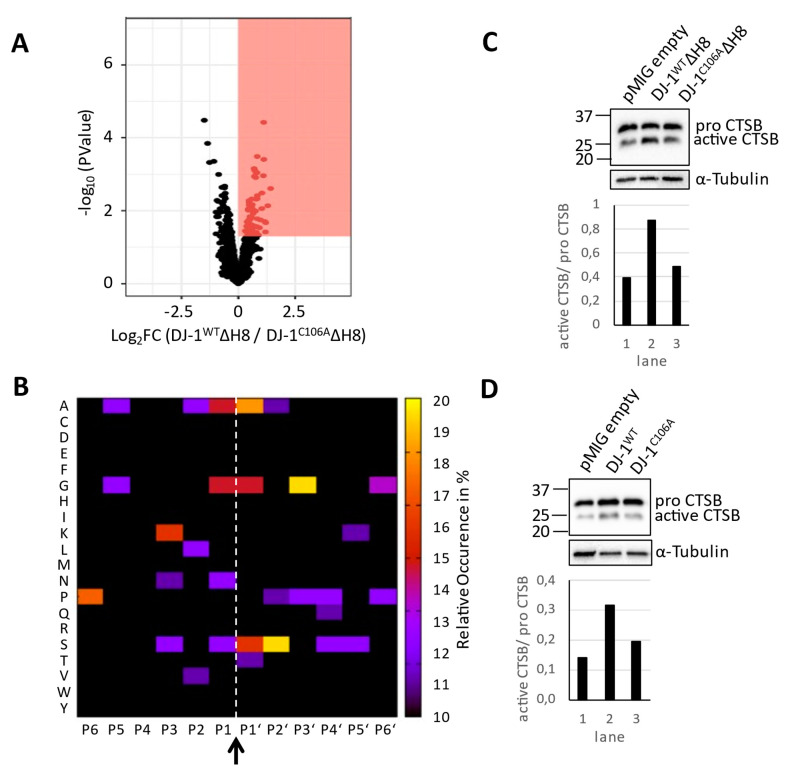
**Degradome analysis:** (**A**) Volcano plot representation of the TAILS experiment of DJ-1^WT^ΔH8 and DJ-1^C106A^ΔH8 overexpressing cells following LIMMA analysis, red box: proteins with higher abundance in DJ-1^WT^ΔH8 cells and p_limma_ < 0.05 (**B**) Heatmap of the occurrence of amino acids in each position, P6-P6′, of peptides with higher abundance in DJ-1^WT^ΔH8 cells following LIMMA analysis relative to the natural abundance levels of amino acids in humans (arrow and dashed line highlight the hydrolysis site, P1 to P6 were derived bioinformatically) (**C**,**D**) Cathepsin B (CTSB) Immunoblots with α-Tubulin as loading control (upper panels) and quantification of the amount of active CTSB (lower panels).

To identify differentially abundant peptides, the LIMMA package from R was used. As an initial step to identify enriched or underrepresented cleavage sites in connection with overexpression of different DJ-1 types, we considered N-terminal peptides with a non-adjusted *p*-value < 0.05. As shown in Figure 2A, we detected 71 peptides enriched in DJ‑1^WT^ΔH8 overexpressing cells as compared to DJ-1^C106A^ΔH8 overexpressing cells that fulfill this criterion. However, none of these peptides fit to the previously described sequence specificity of DJ-1: V↓A, valine in P1 and alanine in P1‘ (Supplementary Table S1, sheet “enriched cleavage sites”). A summarized cleavage site motif is shown in Figure 2B, which shows the occurrence of amino acids of differentially abundant cleaved peptides. We note that P2 mostly comprises aliphatic residues, whereas P1 and P1′ are predominantly composed of small amino acids. This motif is reminiscent of cysteine cathepsins; e.g., cathepsins B and L [40]. To investigate a potential link to lysosomal biology we probed for Cathepsin B (CTSB) activity by immunoblot. Cathepsin B is a potent lysosomal protease whose activation includes removal of its 62 residue N-terminal domain. We verified a higher amount of activated CTSB in DJ‑1^WT^ΔH8 overexpressing cells compared to DJ-1^C106A^ΔH8 or empty vector control cells (Figure 2C). Analysis of TAILS peptides confirmed significantly more Cathepsin B propeptide cleavage in DJ‑1^WT^ΔH8 relative to DJ-1^C106A^ΔH8 overexpressing cells (Figure S2). A higher amount of the CTSB active form was also found in cells overexpressing full length DJ-1^WT^ compared to DJ-1^C106A^ or empty vector control cells (Figure 2D). In conclusion, the affected degradome of DJ-1^WT^ΔH8 overexpressing cells is dominated by cysteine cathepsin-type proteolysis when compared to the degradome of DJ-1^C106A^ΔH8 overexpressing cells. CTSB activation upon DJ-1 overexpression is dependent on C106 but independent of H8.

### 3.3. Systemic Impact of DJ-1 ΔH8 on the Cellular Proteome

**Figure 3:**
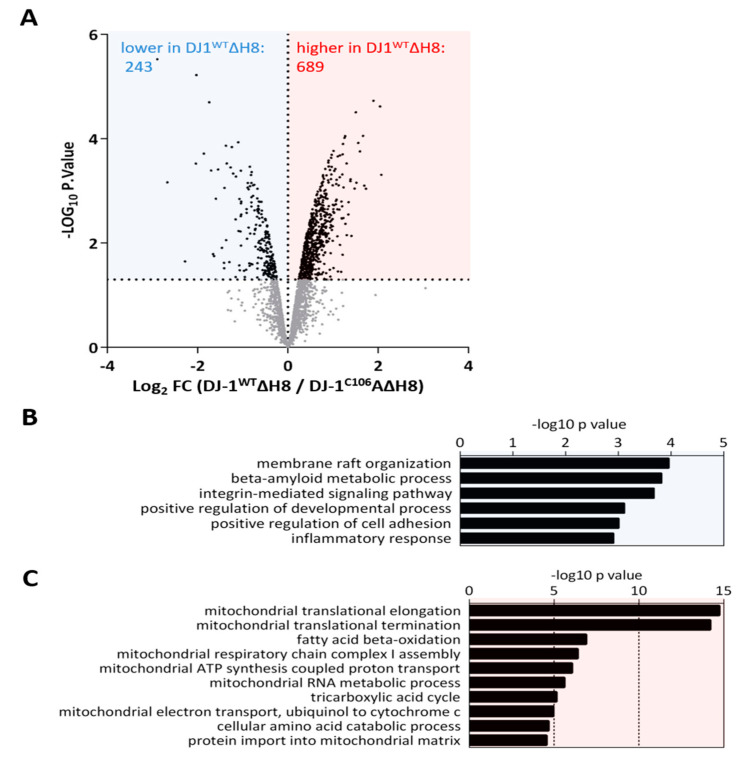
**Systemic effect of DJ-1ΔH8 on the proteome of neuron-like cells:** (**A**) Volcano plot representation of proteins identified in the quantitative proteome comparison of DJ-1^WT^ΔH8 and DJ-1^C106A^ΔH8 overexpressing differentiated SH-SY5Y cells following LIMMA analysis of three independent replicates, blue box: proteins significantly higher and red box: proteins significantly lower in DJ-1^WT^ΔH8 (log2 FC ≤ −0.58 or ≥ 0.58) in two of three experiments and *p*-value ≤ 0.05) (**B**) Gene Ontology Biological Processes overrepresented by proteins significantly lower in DJ-1^WT^ΔH8 overexpressing cells (**C**) Gene Ontology Biological Processes overrepresented by proteins significantly higher in DJ-1^WT^ΔH8 overexpressing cells.

Some of the observed altered cleavage events that could not have been generated by cysteine cathepsins, might be explained by a systemic effect of DJ-1^WT^ΔH8 overexpression. To approach this question, we conducted a quantitative proteome comparison of cells overexpressing DJ-1^WT^ΔH8 and cells overexpressing DJ-1^C106A^ΔH8 (see methods). A total number of 2334, 2933 and 4421 proteins were identified in the first, second and third experiment, respectively. An overlap of 2053 proteins was consistently found in all three experiments (Figure S3A). DJ-1^WT^ΔH8/ DJ-1^C106A^ΔH8 normalized ratios showed a normal distribution in all three experiments (Figure S3B). Alterations of protein abundances were calculated as log2 fold change (FC) values of DJ-1^WT^ΔH8/DJ-1^C106A^ΔH8 normalized ratios. To define proteins with altered abundance, LIMMA was used. Only proteins with an abundance change of more than 50% (FC> |log2(1.5)|) in two of three experiments and with a non-adjusted *p*-value ≤ 0.05 were considered to be significantly altered (Supplementary Table S2). We found 689 proteins with significantly higher abundance and 243 proteins with significantly lower abundance in DJ-1^WT^ΔH8 overexpressing cells compared to DJ-1^C106A^ΔH8 overexpressing cells (Figure 3A). To interpret the pronounced effect of DJ-1^WT^ΔH8 on the proteome of neuron-like cells, all proteins significantly altered in abundance were clustered into gene ontology biological processes (GO BPs). The most significantly enriched GO BP terms are shown in Figure 3B,C. Proteins with a lower abundance in DJ-1^WT^ΔH8 are involved e.g., in membrane raft organization, beta amyloid metabolism, and integrin mediated signaling (Figure 3B). Proteins enriched in DJ‑1^WT^ΔH8 primarily play a role in mitochondrial processes such as mitochondrial transcription, mitochondrial translation, respiration, and mitochondrial metabolic processes including fatty acid beta oxidation and mitochondrial RNA metabolism. Many studies have shown a role of DJ-1 in maintaining proper mitochondrial function (reviewed in [41]). Here we show that C-terminally truncated DJ-1 lacking H8 affects mitochondrial processes in a C106-dependent manner as well.

**Figure 4:**
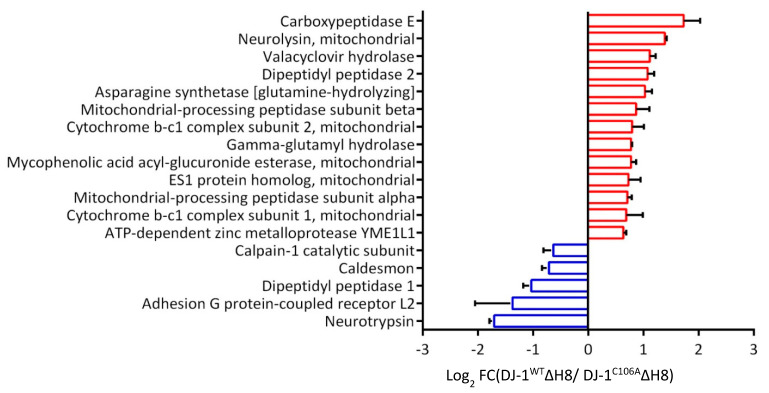
**Effect of DJ-1**Δ**H8 overexpression on the abundance of other proteases:** Proteases with significantly altered abundance in the quantitative proteome comparison of DJ-1^WT^ΔH8 and DJ-1^C106A^ΔH8 overexpressing differentiated SH-SY5Y cells (log2 FC ≤ −0.58 or ≥ 0.58) in two of three experiments and *p*-value ≤ 0.05).

For the investigation of a putative indirect effect of DJ-1^WT^ΔH8 on the degradome that we observed in the TAILS experiment, we reviewed the proteins significantly higher or lower in the quantitative proteome comparison in detail. We found several proteases with altered abundance (Figure 4). Seven mitochondrial proteases, such as Neurolysin and Mitochondrial processing peptidase subunit beta, increased in DJ-1^WT^ΔH8 overexpressing cells. Also, two lysosomal proteases, namely Dipeptidyl peptidase 1 and Gamma-glutamyl hydrolase, showed higher abundance in DJ-1^WT^ΔH8 overexpressing cells. In conclusion, the observed effect on the cellular degradome can partially be explained by the altered abundance of other proteases, including non-cysteine-type lysosomal proteases and mitochondrial proteases.

### 3.4. Protective Effect of DJ-1 on Protein Glycation

**Figure 5:**
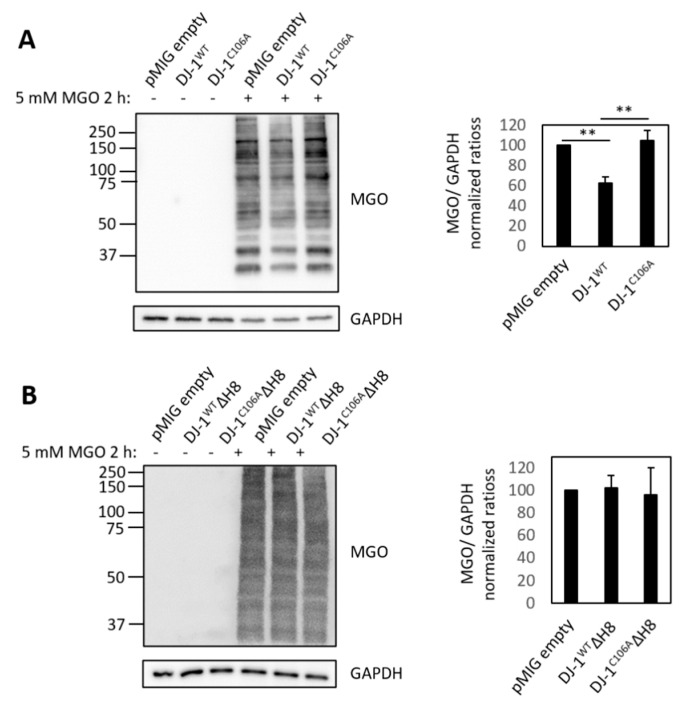
**Effect of DJ-1 on protein glycation:** Effect on protein glycation by overexpression of (**A**) full length DJ-1 variants DJ-1^WT^ and DJ-1^C106A^ and (**B**) C-terminally truncated DJ-1 variants; left: Immunoblots of whole cell lysates of untreated and MGO treated cells expressing different DJ-1 variants with anti-MGO antibody and GAPDH as loading control: right: quantification of signal intensity of MGO-modified proteins relative to empty vector control (pMIG empty) with ImageJ of three independent experiments for full length variants and two independent experiments for C-terminally truncated variants (** *p* ≤ 0.01 by two tailed t-test on independent groups).

A few years ago, DJ-1 deglycase activity was reported [14]. The authors suggested that deglycation constitutes the primary cell-physiological function of DJ-1. However, this concept has been challenged [42,43]. Using the in vitro model system of differentiated SH SY5Y cells, we probed for the potential deglycation properties of the four different DJ-1 variants. Differentiated SH-SY5Y cells were exposed to 5 mM methylglyoxal (MGO) for 2 h in culture. Immunoblots of whole protein extracts with an anti-MGO antibody showed a significant decrease of protein glycation in cells overexpressing DJ-1^WT^ compared to empty vector control cells (Figure 5A), which was abolished in the DJ-1^C106A^ mutant. These results confirm a C106‑dependent protective effect of DJ-1 on protein glycation in neuron-like cells. In contrast, protein glycation was not decreased in DJ-1ΔH8 overexpressing cells compared to empty vector control cells (Figure 5B). Our findings corroborate that DJ-1 protects proteins from glycation and we show that the C-terminal Helix 8 (H8) is essential for this protective effect.

## 4. Discussion

DJ-1 plays a role in tumor progression (reviewed in [44]) and DJ-1 loss-of-function is associated with autosomal early-onset Parkinson’s disease [1]. DJ-1 protects cells from oxidative stress [7], but its precise function remains elusive. The aim of the present study is to further our understanding of the cell-physiological roles of DJ-1 and their dependence on helix 8, which supposedly acts as a propeptide. Due to the suggested role of DJ-1 in neurodegenerative diseases, we employed an in vitro model of post-mitotically differentiated, neuron-like cells.

While our N-terminomic analysis revealed differences in the degradome between DJ-1^WT^ΔH8 and DJ‑1^C106A^ΔH8 overexpressing cells, we did not find quantitatively affected N‑termini that match the sequence specificity of proteolytically active DJ-1 (V↓A) that was determined in vitro before [17]. We note that this finding does not refute DJ-1 acting as an endoprotease in cellulo. It is possible that putative DJ-1 substrates were absent in differentiated SH-SY5Y cells. Furthermore, it is possible that DJ-1 cleavage products are prone to rapid degradation, hence escaping detection by N-terminomics.

Independent of direct DJ-1 substrates, we found increased levels of several other, mainly mitochondrial and lysosomal, proteases and observed a pronounced, yet indirect impact of DJ‑1 on cysteine cathepsin-type proteolysis. This impact depends on the presence of C106 but is independent of H8. Of note, we do not postulate that DJ-1 directly activates Cathepsin B but consider this an indirect, yet dominant effect whose mechanistic underpinning remains beyond the findings of the present study. Our findings are in line with previous reports that link the cell-physiological function of DJ-1 to lysosomal biology: Gao and colleagues reported increased autophagy upon DJ-1 overexpression [45] and other groups showed reduced autophagy upon loss of DJ-1 [46,47]. The link between DJ-1 and autophagy awaits further mechanistic clarification.

The quantitative proteome comparison of DJ-1^WT^ΔH8 and DJ-1^C106A^ΔH8 overexpressing cells showed an increase of mitochondrial proteins in DJ-1^WT^ΔH8. The protective function of DJ-1 on mitochondria and the reduction of reactive oxygen species was observed before in several studies (reviewed in [41]). Proteins more abundant in DJ-1^WT^ΔH8 overexpressing cells are involved in biological processes such as mitochondrial translation, complex I assembly and transport into mitochondria, which are important for mitochondrial biogenesis. Hence it is conceivable that DJ-1 is not only linked to the maintenance but also to the biogenesis of mitochondria.

Interestingly, we observed an effect on mitochondrial and lysosomal biology when overexpressing the truncated form DJ-1^WT^ΔH8. DJ-1 exists as a homodimer in cells, which is its expected active form. Based on its crystal structure, a novel mode of dimerization mediated by C-terminal helix H8 was suggested [21]. It remains to be determined whether DJ-1 dimerization is dependent on H8 or whether it may also function as a monomer. We observed lower protein amounts of the overexpressed truncated versions in comparison to the DJ-1 full length versions despite an equal expression of GFP in the bicistronic system. This could mean that the half live of DJ-1ΔH8 is shorter than that of full length DJ-1.

The accumulation of glycated proteins and lipids, which represent so-called “advanced glycation end products” (AGE), is a feature of neurodegenerative diseases. Proteins and lipids become glycated by the exposure to reducing sugars such as MGO. The accumulation of AGE may contribute to the development of neurodegenerative disease [48]. A protective effect of DJ-1 against protein glycation has been shown in yeast, bacteria [49] and human keratinocytes [50]. Whether DJ-1 detoxifies free MGO by acting as a glyoxalase [12,42,51] or removes adducts formed by MGO on proteins by acting as a deglycase [14,52] is a matter of debate. Here we confirm a C106-dependent protective effect of DJ-1 on protein glycation in neuron-like cells and show that in our cellular system H8 is essential for this effect.

In summary, we used neuron-like SH-SY5Y cells to study the physiological role(s) of DJ-1ΔH8. We were unsuccessful in identifying DJ-1 proteolytic substrates, but our findings strengthen the impact of DJ-1 on lysosomal and mitochondrial biology, as well as its protective role against protein glycation. All of these activities depend on the presence of cysteine 106, while the C-terminal Helix 8 (H8) is dispensable for the link to lysosomal biology.

## Data Availability

Raw Data of mass spectrometry experiments is accessible via MassIVE: ftp://MSV000086626@massive.ucsd.edu (accessed on 10 February 2021); reviewer password “ImpactofDJ1”.

## Supplementary Materials

The following are available online at https://www.mdpi.com/2073-4409/10/2/404/s1, Figure S1: Cell system. Figure S2: Quantitative proteome comparison of differentiated DJ-1^WT^ΔH8 and DJ-1^C106A^ΔH8 overexpressing SH-SY5Y cells. Table S1: 2020-11-23, Table S2: 2020-11-30.
