# Supplementary material for: Impact of DJ-1 and Helix 8 on the Proteome and Degradome of Neuron-Like Cells
Source: Cells. 2021 Feb 16;10(2):404. doi: 10.3390/cells10020404 (PMC7920061; doi:10.3390/cells10020404)
Supplement: Supplementary file 1 [file cells-10-00404-s001.zip › SupplementaryFiguresfinal.pptx]

## Slide 1
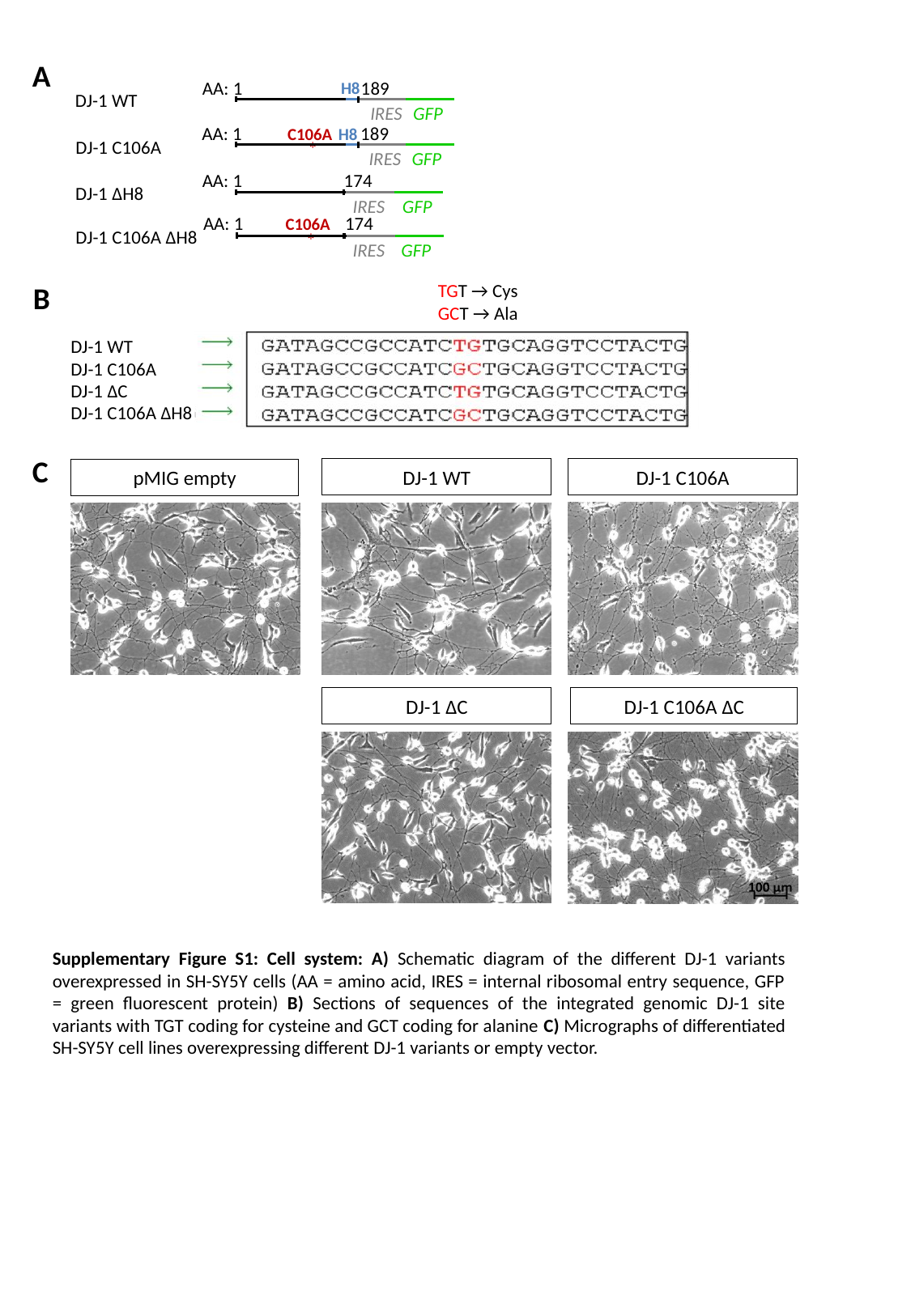

A
AA: 1 189
H8
DJ-1 WT
IRES
GFP
AA: 1 189
C106A
H8
DJ-1 C106A
*
IRES
GFP
AA: 1 174
DJ-1 ΔH8
IRES
GFP
AA: 1 174
C106A
DJ-1 C106A ΔH8
*
IRES
GFP
B
TGT → Cys
GCT → Ala
DJ-1 WT
DJ-1 C106A
DJ-1 ΔC
DJ-1 C106A ΔH8
C
DJ-1 WT
DJ-1 C106A
pMIG empty
DJ-1 ΔC
DJ-1 C106A ΔC
Supplementary Figure S1: Cell system: A) Schematic diagram of the different DJ-1 variants overexpressed in SH-SY5Y cells (AA = amino acid, IRES = internal ribosomal entry sequence, GFP = green fluorescent protein) B) Sections of sequences of the integrated genomic DJ-1 site variants with TGT coding for cysteine and GCT coding for alanine C) Micrographs of differentiated SH-SY5Y cell lines overexpressing different DJ-1 variants or empty vector.

## Slide 2
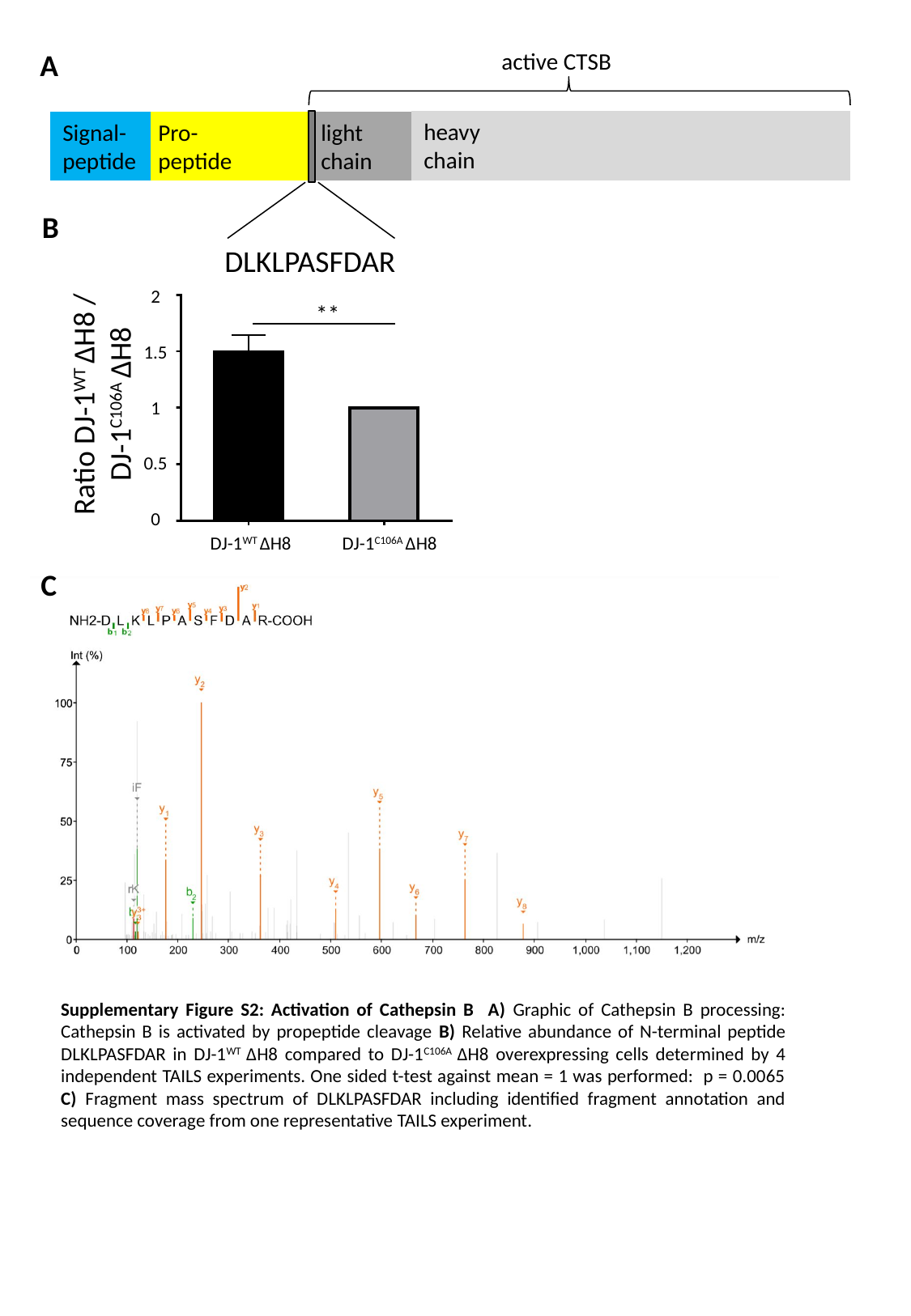

active CTSB
A
heavy
chain
Signal-
peptide
Pro-
peptide
light
chain
B
DLKLPASFDAR
2
**
1.5
Ratio DJ-1WT ΔH8 /
 DJ-1C106A ΔH8
1
0.5
0
DJ-1WT ΔH8
DJ-1C106A ΔH8
C
Supplementary Figure S2: Activation of Cathepsin B A) Graphic of Cathepsin B processing: Cathepsin B is activated by propeptide cleavage B) Relative abundance of N-terminal peptide DLKLPASFDAR in DJ-1WT ΔH8 compared to DJ-1C106A ΔH8 overexpressing cells determined by 4 independent TAILS experiments. One sided t-test against mean = 1 was performed: p = 0.0065 C) Fragment mass spectrum of DLKLPASFDAR including identified fragment annotation and sequence coverage from one representative TAILS experiment.

## Slide 3
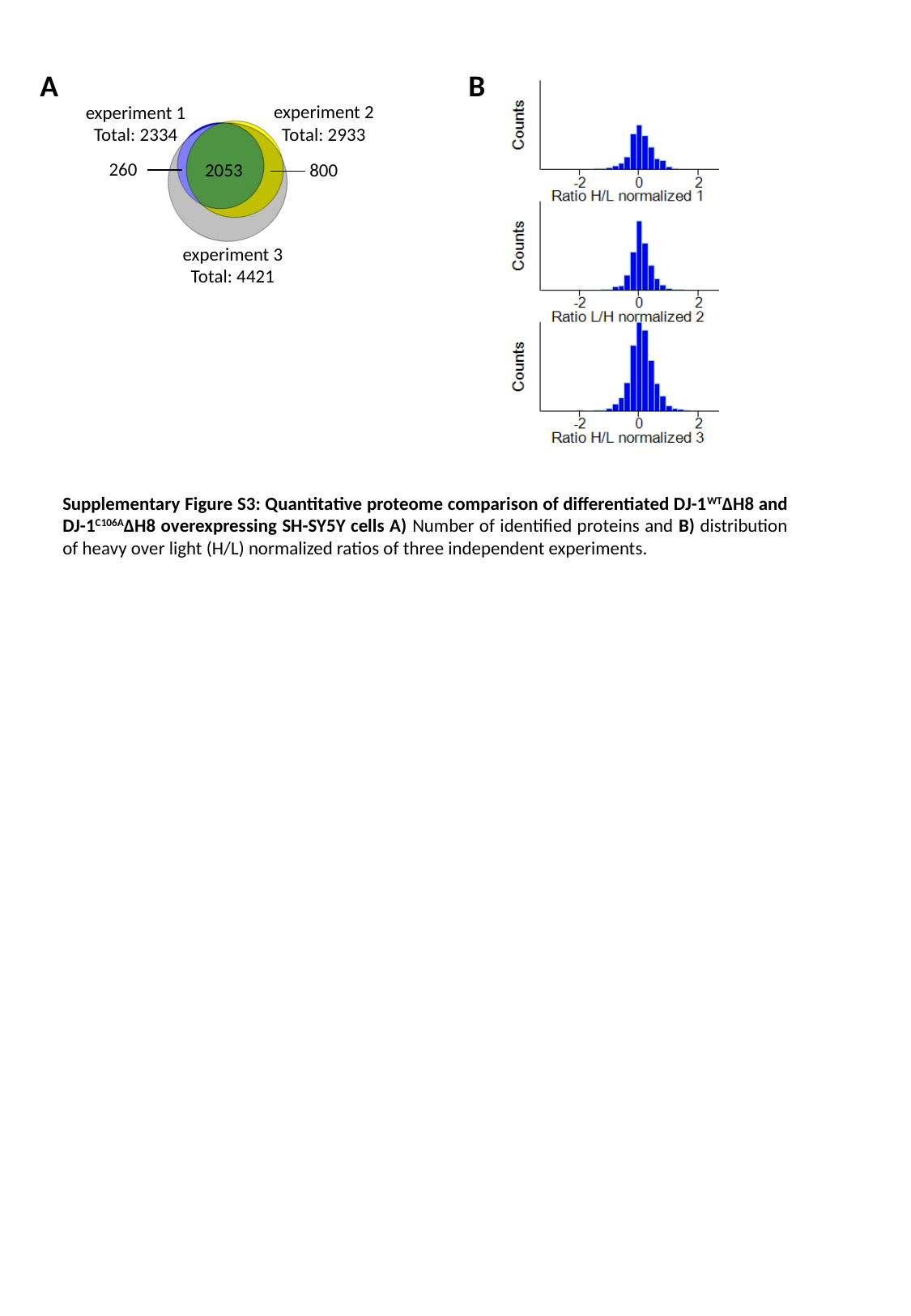

A
B
experiment 2
Total: 2933
experiment 1
Total: 2334
260
800
2053
experiment 3
Total: 4421
Supplementary Figure S3: Quantitative proteome comparison of differentiated DJ-1WTΔH8 and DJ-1C106AΔH8 overexpressing SH-SY5Y cells A) Number of identified proteins and B) distribution of heavy over light (H/L) normalized ratios of three independent experiments.
